# Abstract of Proceedings of the Buffalo Medical Association

**Published:** 1867-04

**Authors:** T. M. Johnson

**Affiliations:** Sec’y.


ART. III—*Abstract of Proceedings of the Buffalo Medical Association.*

TUESDAY EVENING, March 5, 1867.

The meeting was called to order by the President at the usual hour. Present—Drs. Gould, Jansen, Little, Brown, Wyckoff, Miner, Wetmore, Kamerling, Abbott, Rochester, Gay, Samo, Trowbridge, Greene, Strong, Congar, Cronyn and Johnson.

On motion of Dr. Miner the reading of the minutes of the last meeting was dispensed with.

DR. MINER stated that Dr. J. R. Lothrop, who was expected to read an essay this evening, was too ill to attend the meeting.

Drs. Charles S. Sheldon, C. A. Nichell and Charles P. Schuyler, were, by vote of the Association, invited to attend its meetings until the next meeting of the Erie County Medical Society.

There being no other business before the meeting, reports on prevailing diseases were called for. Influenza, capillary pneumonia and bronchitis were reported as prevailing.

DR. ROCHESTER remarked that he had never seen influenza prevail with so much of an epidemic character as at present, and said that the disease is not confined to children and young persons, but many old people are suffering from it. Has seen many cases complicated with pneumonia. Would call attention to the fact that influenza is said to be often followed by epidemic cholera.

Dr. R. also stated that whooping cough, of a severe type, is prevailing at Black Rock, with an unusual number of fatal cases.

DR. MINER had seen two children with all the symptoms of severe bronchitis with dyspnœa and periodic attacks of asthmatic or croupy breathing. Was not fully satisfied of the nature of the disease, but regarded it as extensive, not very acute bronchitis; they were very sick, and their parents thought they would die. Perhaps influenza is a better name, and more completely represents the peculiar appearances of the disease. Had not hitherto considered influenza a disease of much importance, but in the cases mentioned the patients were really quite sick. Had not seen many of similar nature.

Dr. Rochester remarked that he had seen cases in which the dyspnœa was as severe as in croup. Influenza sometimes seems to affect the air passages principally. Many times it seems to affect the whole system, producing great depression. The rapid and marked prostration seems a part of the malady. It prevails with all classes and among the old and young. It is not exclusively a disease of the air passages. Have found that in these cases stimulus is required. Quinine, carbonate of ammonia, and sometimes alcoholic stimulus is necessary. This treatment is better than that by pulv. doveri and the ordinary or less active plan. Stimulating treatment is often necessary from the outset.

Dr. Wyckoff had seen many cases of the disease, in most of which there had been great dyspnœa and prostration. Many cases are complicated with bronchitis, followed sometimes by consolidation of some portion of the lung. Had also seen many cases where diarrhœa also prevailed in conjunction with the disease. Had found carbonate of ammonia, the preparations of bark, etc., necessary in a majority of cases.

Dr. Strong reported a case of death from tinct. opii. Was called at eight o'clock in the morning to see a child seven days old, to whom its parents had administered one drop of tinct. opii six hours before, and repeated the dose an hour and a half later. Found it almost lifeless. It had reviving and sinking spells and lived twenty-eight hours after taking the first dose. This seems a very small dose to be attended with fatal results. It will be remembered that I reported a case at a former meeting in which a child a year old, took with impunity nearly a drachm of the tincture.

Dr. Gould saw a case in which a child two weeks old took six drops and died. Once gave to a woman a six gr. pill of opium every hour for twelve successive hours with no ill effects.

Dr. Rochester would inquire of Dr. Strong if he considered two drops of tinct. opii a small dose for a child six or seven days old, and if he would give such a dose?

Dr. Strong replied that he would not give such a dose. Would not give tinct. opii to so young a child.

Dr. Miner remarked that tinct. opii, becomes much stronger by long standing; that in many instances, even when the bottle containing it appears well corked, it at length becomes concentrated to fluid opium; this should be remembered when families use this medicine. The tincture as furnished by druggists undoubtedly varies greatly in strength.

Dr. C. C. Wyckoff was elected to read an essay at the next regular meeting.

Adjourned. T. M. Johnson, Sec'y.

